# Supplementary figures and images for: The Clinical Utility of Serum Alpha-1-Acid Glycoprotein in Reflecting the Cross-Sectional Activity of Antineutrophil Cytoplasmic Antibody-Associated Vasculitis: A Single-Centre Retrospective Study
Source: Medicina (Kaunas). 2024 Jul 26;60(8):1212. doi: 10.3390/medicina60081212 (PMC11356503; doi:10.3390/medicina60081212)

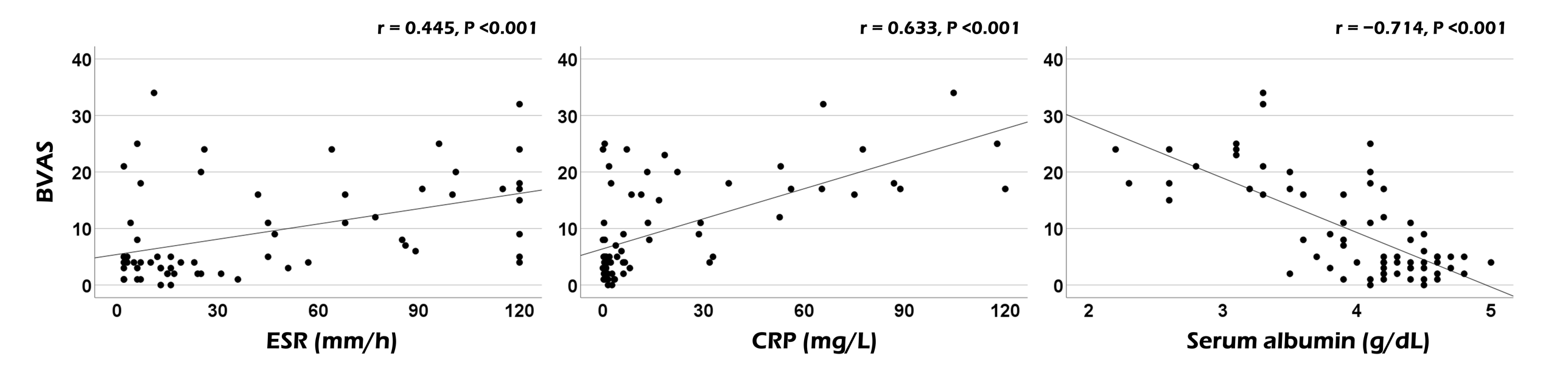

Supplement: Supplementary file 1 [file medicina-60-01212-s001.zip › medicina-3092955-supplementary/FIGURE S1(medicina-3092955)(1stREVISION).tif]

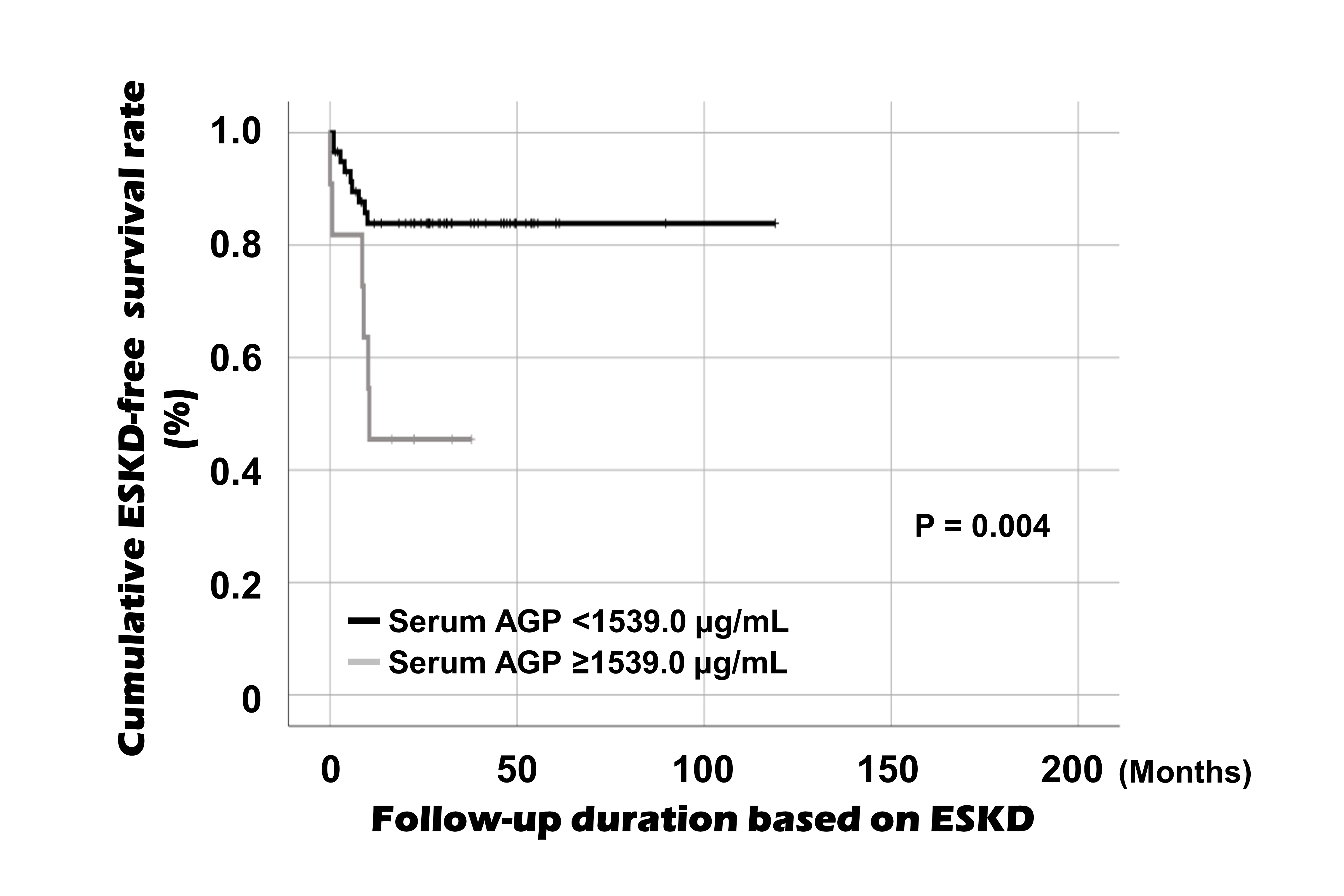

Supplement: Supplementary file 1 [file medicina-60-01212-s001.zip › medicina-3092955-supplementary/FIGURE S2(medicina-3092955)(1stREVISION).tif]
